# Methamphetamine Use and Emergency Department Utilization: 20 Years Later

**DOI:** 10.1155/2017/4050932

**Published:** 2017-08-17

**Authors:** John R. Richards, Sheiva Hamidi, Connor D. Grant, Colin G. Wang, Nabil Tabish, Samuel D. Turnipseed, Robert W. Derlet

**Affiliations:** Department of Emergency Medicine, University of California Davis Medical Center, Sacramento, CA, USA

## Abstract

**Background:**

Methamphetamine (MAP) users present to the emergency department (ED) for myriad reasons, including trauma, chest pain, and psychosis. The purpose of this study is to determine how their prevalence, demographics, and resource utilization have changed.

**Methods:**

Retrospective review of MAP patients over 3 months in 2016. Demographics, mode of arrival, presenting complaints, disposition, and concomitant cocaine/ethanol use were compared to a 1996 study at the same ED.

**Results:**

638 MAP-positive patients, 3,013 toxicology screens, and 20,203 ED visits represented an increase in prevalence compared to 1996: 461 MAP-positive patients, 3,102 screens, and 32,156 visits. MAP patients were older compared to the past. Mode of arrival was most frequently by ambulance but at a lower proportion than 1996, as was the proportion of MAP patients with positive cocaine toxicology screens and ethanol coingestion. Admission rate was lower compared to the past, as was discharge to jail. The proportion of MAP patients presenting with blunt trauma was lower compared to the past and higher for chest pain.

**Conclusion:**

A significant increase in the prevalence of MAP-positive patients was found. Differences in presenting complaints and resource utilization may reflect the shifting demographics of MAP users, as highlighted by an older patient population relative to the past.

## 1. Introduction

At present, amphetamines may be legally prescribed for treatment of narcolepsy and attention deficit hyperactivity disorders. Amphetamine and its derivatives, such as methamphetamine (MAP), were first synthesized in the early 20th century and marketed as bronchodilators [1]. However, after their introduction, these drugs were soon used for myriad unrelated conditions, such as for weight loss and to increase wakefulness. Legal availability of amphetamines led to widespread use until being designated as controlled Schedule II drugs in 1970. After this, MAP faded from popularity until the late 1980s, where it reappeared in the western United States and Hawaii [2]. In 1989, Derlet and coworkers published the first study of patients with MAP toxicity in the emergency department (ED) and found that agitation, hallucinations, suicidal behavior, and chest pain were the most common presenting complaints [3]. During the 1990s, MAP use continued to grow in the Northwest and Southwest. By the millennium, MAP use had become entrenched in the Midwest as well and to a lesser degree in the South, Northeast, and Mid-Atlantic states [4]. During this period, authors of the National Survey on Drug Use and Health (NSDUH) estimated that MAP use increased from approximately 2% of the adult population in 1994 to 5% over the following decade [5]. Patients abusing MAP present to the ED for acute cardiovascular, psychiatric, toxicologic, neurologic, and traumatic disorders [6]. Richards and associates published the first study of ED utilization by MAP users versus nonusers in 1996 and found significantly higher rates of arrival by ambulance and admission to the hospital [7]. The United States Drug Abuse Warning Network (DAWN) began monitoring MAP-associated ED visits in 1995 and reported 11,002 visits in 1996 [8]. From the last published DAWN report in 2014, there were 102,961 MAP-associated ED visits in 2011 [9].

Methamphetamine use continues to be a significant problem domestically and worldwide. From the most recently published NSDUH in 2015, approximately 897,000 people aged 12 or older were current users of MAP, a substantial increase from 569,000 in the prior year [10]. From a global perspective, there are over 24 million estimated regular MAP users [10]. The United States has consistently reported the largest amount of MAP seizures by law enforcement each year, followed by East and Southeast Asia, where these have quadrupled between 2009 and 2014 [11–13]. Eastern Europe, Russia, Oceania, and the Middle East have also experienced a growing number of MAP users during recent years [12, 14–16]. As the prevalence of MAP use continues to rise throughout the country, we repeated the original 1996 study of ED utilization 20 years later to determine if this was an institutional trend as well and to further characterize this patient subgroup.

## 2. Methods

This study was performed over a three-month period from May to August 2016 at the University of California, Davis Medical Center, an urban, academic Level I trauma center with an annual ED census of 80,000 visits. This ED serves a population of 500,000 within the Sacramento city limits and 1.6 million in the surrounding area. The hospital also serves as a tertiary referral center for Northern and Central California and is the de facto public hospital, providing care for a significant number of uninsured and/or dispossessed patients, as well as those brought in by law enforcement from the street, jails, prisons, and detention centers. A retrospective review of patients presenting to the ED with MAP-positive urine toxicology screens was undertaken. Collected data included demographics, mode of arrival, presenting complaints, disposition, and ethanol level, which was then compared to a similar study performed at the same ED 20 years previously. The electronic medical record of each patient was accessed, and data were recorded on a standardized form by the study authors. Interrater reliability was not evaluated. The qualitative urine toxicology screen was performed using a UniCel DxC 800 Synchron (Beckman Coulter Inc., Brea, California) to detect MAP and other drugs of abuse. There is no standardized protocol in place at our ED for ordering toxicology screens, with the exception of trauma patients admitted to the hospital and patients on 72-hour psychiatric holds. Otherwise, the decision to obtain toxicology screens is at the discretion of the treating clinician. Data were entered into Excel (version 14, Microsoft, Redmond, Washington) and analyzed with Stata (version 12, StataCorp, College Station, Texas). Statistical analysis was performed using chi-square and unpaired Student's* t*-tests. Results are reported as mean ± standard deviation (SD) unless otherwise stated. Statistical significance is assumed at a level of *P* ≤ 0.05. This study was approved by the institutional review board at our institution.

## 3. Results

For the three-month period in 2016, a total of 638 patients were identified as MAP-positive out of 3,013 total urine toxicology screens and 20,203 ED patient visits. In the 1996 study over a six-month period, there were 461 MAP-positive patients from a total of 3,102 toxicology screens and 32,156 ED visits This represented a significant increase in both the prevalence of MAP-positive toxicology screens in 2016 compared to 1996 (21.2% versus 14.9%) and the proportion of MAP-positive patients presenting to the ED (3.2% versus 1.4%). Differences in demographics, ED arrival, disposition, and concomitant ethanol and cocaine use are shown in Table 1. Methamphetamine patients were significantly older compared to the past (41.6 ± 12.6 versus 34.9 ± 8.5 years), but there was no gender difference observed. The most frequent mode of arrival was by ambulance but at a lower proportion compared to the past (52% versus 69.2%). Racial comparison revealed no significant differences, with the majority of users identified as Caucasian.

With regard to medical insurance status, a higher proportion of MAP-positive patients in 2016 had state, federal, or commercially funded medical insurance compared to 1996. A lower proportion of MAP-positive patients in 2016 had positive cocaine toxicology screens than in 1996 (4.4% versus 7.4%) as well as ethanol coingestion (12.4% versus 20%). Admission rate in 2016 was significantly lower compared to the past (41.2% versus 58.1%) as was discharge directly to jail (1.3% versus 8.9%). Presenting complaints between the time periods are compared in Table 2. The proportion of MAP-positive patients presenting with blunt trauma was significantly lower than the past (12.2% versus 33%, *P* = 0.0001) and higher for chest pain (16% versus 7.8%, *P* = 0.0001).

## 4. Discussion

At present, this study is the first in which MAP prevalence and user characteristics have been compared at the same medical treatment facility over a two-decade period of time. Our finding of increasing MAP prevalence parallels findings from multiple national and worldwide databases. Attempts to hinder domestic MAP production by outlawing specific chemical precursors such as phenylacetone, pseudoephedrine, and ephedrine has resulted in a decrease in domestic MAP production by 56 percent from 2010 to 2015 [17]. However, the United States Drug Enforcement Agency (DEA) reports that Mexico has now become the major supplier of MAP [17, 18]. According to the 2016 National Drug Threat Survey, almost one-third of responding law enforcement agencies reported MAP as the greatest drug threat in their areas, specifically in the Southwest, West Central, West Coastal, and Southeast regions, and it is the drug that most contributes to violent crime [17]. Decreasing drug price and increasing purity may be contributing factors to the recent increase in MAP prevalence: DEA analysis of domestic MAP purchases from 2007 through 2015 revealed that the price per gram of pure MAP decreased by 57% from $152 to $66, while the purity increased from 56% to 92% [17].

There seems to be no indication that the trend of increasing MAP prevalence will reverse in the near future. Data from Quest Diagnostics (Madison, New Jersey), which performs screening tests for drugs of abuse for employers and hospitals, indicates a steady rise in the prevalence of positive tests for MAP over the past 5 years [19]. Information from the Treatment Episode Data Set (TEDS) from 2003 to 2013 indicates a rise in the number of people admitted for MAP treatment in both the state of California and nationwide [20, 21]. In San Diego, California, MAP prevalence in adult arrestees increased from 28% in 2000 to 41% in 2013 for males and from 29% to 46% in females [22]. In Sacramento County, where our study was conducted, MAP was detected in 27% of patients admitted to the county-funded Mental Health Treatment Center's Intake Stabilization Unit in 2015, and ED visits for MAP-related complaints increased 85% versus 13% for all patients over a 3-year period [23]. An analysis of MAP-screening test positivity by zip code showed Sacramento County to have a rate of 1.7%–3.8%, which represented the highest tier recorded statewide, along with Los Angeles and San Diego [19].

There have been a small number of studies focusing on MAP use and ED care at our institution [3, 24–31]. The first appeared in 1989, when Derlet and colleagues published a retrospective study of MAP patients presenting to our ED [3]. Over a 6-month period in 1987, 127 MAP-positive patients out of a total of 18,510 were identified. This represents prevalence of 0.7% compared to our findings of 1.4% in 1996 and 3.2% in 2016. Gender proportion (65% male) was similar to 1996 and 2016, and 54% were aged 30 years and older. Admission rate was 19%, compared to 58.1% in 1996 and 41.2% in 2016. Psychiatric hold and admission rate was 49% versus 13.7% in 1996 and 35% in 2016. Patients presenting with trauma were not included in their study, but two-thirds were brought to the ED for altered mental status or being found unresponsive. In the next few studies, Richards and associates examined the treatment of MAP-induced agitation and investigated the association of rhabdomyolysis, tooth wear, and acute coronary syndrome (ACS) with MAP use [24, 26–28].

Schermer and Wisner investigated the association of MAP with trauma from 1989 to 1994 at our institution and reported that MAP prevalence in 18,004 trauma patients increased from 7.4% to 13.4% compared to cocaine rates (5.8% to 6.2%) and ethanol (43% to 35%) [25]. Similar to our findings, MAP-positive patients were most likely to be male and Caucasian and to be involved in motor vehicle and motorcycle collisions. This was in contradistinction to cocaine-positive patients, who were most commonly non-Caucasian and were injured by assaults, gunshot, or stab wounds. In another trauma-based study of 10,663 subjects at our hospital from 2002 to 2006, London et al. determined that minimally injured (Injury Severity Score < 9) MAP-positive patients incurred more cost and utilized more hospital resources than non-MAP patients [29]. Demographics and racial profile were similar to our study and the aforementioned trauma study years earlier, and mechanism of injury was predominantly blunt trauma. Insurance status was assessed, and 49% of MAP-positive patients were uninsured, which fell between our findings of 83.5% in 1996 and 27% in 2016. Potential reasons for the increased costs and hospital resource utilization were from the acute effects of MAP, resulting in an unreliable physical examination, tachycardia, and acidosis, suggesting shock from occult injury, and altered mental status raising the possibility of head injury. Patients with MAP toxicity may be hypersomnolent during metabolism of the drug and repletion of monoamines and adenosine triphosphate (ATP), delaying discharge while increasing length of stay [30].

Lee et al. then performed a study from 2004 to 2006 in which 318 MAP patients' self-reporting of their drug use was correlated to their toxicology screen [31]. The authors determined that the self-report rate for MAP-positive patients was 52%, highlighting the importance of toxicology screening in the diagnostic process. A final study was performed between 2009 and 2010 to determine the prevalence of MAP in 1,207 psychiatric patients evaluated in our ED and whether detection of MAP on toxicology screening was associated with involuntary 72-hour holds [32]. The authors reported MAP prevalence of 15%, which was closer to our 1996 findings and lower than 2016. Demographics were similar to our findings, although 41% of MAP-positive patients were uninsured compared to 27% of MAP-negative patients. This rate fell between our findings in 1996 and 2016.

There have been a small number of studies performed at other hospitals which have focused on the utilization of resources by MAP-positive patients. Tominaga et al. studied 212 Hawaiian trauma patients in 2002 and reported MAP prevalence of 27% [33]. Patients who were MAP-positive were most likely to have injuries from motor vehicle collision or blunt force assault rather than from penetrating trauma. Similar to previous studies from our institution, they also had higher length of stay, intensive care unit admissions, and hospital charges [25, 29]. In 2005, Gray and coworkers examined 13,125 patients visits at a major Australian ED and determined MAP prevalence rate of 1.2%, similar to our finding in 1996 [34]. It is important to note that there was no toxicology screening for MAP in their study; rather patient history and judgment of the treating clinician were used instead, and this likely underestimated MAP prevalence. Admission and arrival by ambulance rates were 40% and 32%, respectively, which were lower than our study. Another Australian study of the same period reported similar results [35]. Hendrickson et al. conducted an observational study in 2006 of MAP-related ED visits in an urban, academic Level I trauma center similar to ours and reported a prevalence rate of 2.4%, which fell between our 1996 and 2016 rates [36]. The authors reported similar demographics, an uninsured rate of 50%, and predominance of psychiatric and trauma-related diagnoses. Methamphetamine-related chest pain was reported at a much lower rate (3.1%) than our 1996 (7.8%) and 2016 (16%) rates. This same research group also published a study of MAP-related psychiatric visits at their ED during the same time period [37]. High ED utilization rates were reported in a 2005 to 2007 study of 427 at-risk youths who injected MAP in Vancouver, Canada [38]. A comparative study performed 10 years apart (2004 and 2013) of mental health presentations to EDs in Victoria, Australia, found that the number of patients with concurrent MAP exposure more than doubled from 2.2 to 4.3% [39].

The number of MAP-related ED visits over the past decade has been analyzed using data from DAWN and nearly doubled from 67,954 in 2007 to 102,961 in 2011 [40]. Gender trends were largely unchanged, reflecting male predominance, and there was an increase in MAP patients aged 55 and higher. Admission rate to the hospital during this period was 36%, similar to our 2016 findings. This trend towards older age of MAP use may reflect changing national demographics as a result of declining fertility and rising longevity, referred to as the “Silver Tsunami” [41–43]. Older MAP users may have more experience with drug dosing and frequency of use and may be cannier than younger users [41, 44]. Ageing may also be a factor in the lower rate of blunt trauma from car and motorcycle collisions seen from our analysis; the traffic collision rate has been shown to decline with age as older drivers may be more skilled and have higher risk aversion compared to younger drivers [45]. Chronic MAP use has been associated with ACS and the development of cardiomyopathy, which may also be reflected by a trend towards higher age and ED presentations of chest pain in our study, which more than doubled in 2016 compared to 1996 [28, 46].

The racial distribution observed in our study did not change significantly from 1996 compared to 2016, with predominance of Caucasian MAP users. This trend was also seen in all aforementioned studies and government agency reports regarding demographics of MAP users. This racial distribution does not reflect the most recent census taken for our county, in which 45% of the population are identified as Caucasian, 27% as Hispanic, 14.6% as African American, and 18% as Asian [47]. Although MAP and cocaine are stimulants, the two drugs do not appear to substitute for the each other or share a common user group [25, 48]. One explanation for this is geographic, in that cocaine use is more common in inner cities, whereas MAP use is more common in suburban or rural areas [49]. Insurance status significantly changed in 2016, with only 27% uninsured compared to 84% in 1996. One likely explanation for this is the passage of the Patient Protection and Affordable Care Act of 2010, which resulted in a significant reduction in the number of the uninsured in the United States [50, 51]. Another cause could be increased use of MAP by more patients with employer-based insurance, as the unemployment rate declined over the past few years [52]. This would be consistent with the trend of less coingestion of cocaine and ethanol found in our 2016 data. In theory, a working population may be interested in increased wakefulness and stamina from MAP, enabling longer work hours or several jobs, as opposed to the short-term effect of cocaine or depressant effect of ethanol from alcohol or cocaine.

There were significant differences between 2016 and 1996 with regard to mode of arrival, with decreasing ambulance use and increasing ambulatory and police transport rate. These trends may be linked to the increasing number of MAP-positive patients who presented as walk-ins to the ED for mental health issues such as suicidal ideation and/or attempts, depression, anxiety, and acute psychosis. One reason for this is that Sacramento County withdrew funding for the majority of public mental health clinics and residential treatment facilities several years ago, leaving these patients in a void [53]. Our ED, as well as many others in the area, has taken over this role which has led to serious overcrowding and hallway boarding of ambulatory patients on 72-hour psychiatric holds [53, 54]. Unfortunately, this is not a regional phenomenon [55, 56]. Other factors affecting mode of arrival include transport by police of MAP users with erratic behavior or public intoxication, represented in Table 2 as altered level of consciousness and ingestion. Police are often called in first in lieu of an ambulance for these types of public disturbances. In the past, these patients were often brought directly to jail or publically funded detoxification centers. Now they are most likely to be brought to the ED as a result of California Proposition  47, which was passed in 2014 and reduced many nonviolent crimes to misdemeanors, including possession of MAP [57, 58]. The impact of Proposition  47 can also be seen with discharge directly to jail rates, which significantly decreased compared to 1996 [59].

The decreased hospital admission rate of 2016 compared to 1996 reflects a national trend. The American Hospital Association reported a 7% decline in inpatient admissions per capita between 2008 and 2012 [60]. This may be partly due to the implementation of the two-midnight census rule by the Centers for Medicare and Medicaid Services (CMS) in 2013 and increasing number of Accountable Care Organization (ACO) hospital network affiliations. Increasing numbers of insured patients have more access to outpatient treatment options from the ED rather than inpatient admission. With regard to the declining rate of blunt trauma observed in our study, it is possible that this trend also reflects a state and nationwide trend [61, 62]. The United States has experienced a 31% reduction in its motor vehicle death rate per capita over the past 13 years [61]. There are fewer nonfatal injuries as well when the year 2016 is compared to 1996 [63, 64].

## 5. Limitations

There are several limitations associated with this study. First, it is a retrospective review performed over a span of a few months. As such, it represents a “snapshot” in time of the MAP problem in our ED patient population, and the proportions recorded for the study could possibly have changed over the course of months to years. A longitudinal study would have been the best method to study the trend of MAP use and ED utilization, but this was not possible due to the scale of the project and unavailability of patient data after electronic medical record implementation 10 years after the first study was published. There is no standardized protocol in place at our ED for ordering toxicology screens, with the exception of trauma patients admitted to the hospital and on 72-hour psychiatric holds. Otherwise, the decision to obtain toxicology screens is at the discretion of the treating clinician, which may have led to sampling bias. The area served by our ED is noted to have higher than average levels of MAP consumption and production, and toxicology screens may not be ordered if patients give a history of MAP use. As such, our results may actually have underestimated the prevalence of MAP in our patient population. As it is a single-center study, results may not be applicable to other regional medical centers. No quantitative confirmation of MAP-positive screens was performed, and it is possible that false-positive or false-negative screens may have been recorded. Definitive association between MAP use and each patient's presenting complaint was not possible as the amount of time elapsed between last MAP dose and ED presentation could not be established. The half-life of MAP is up to 12 hours, and toxicology screens may remain positive for up to 72 hours. Therefore, the results of this study only pertain to general consumption of MAP by our ED patients who were tested.

## 6. Conclusions

Compared to 20 years earlier, a significant increase has occurred in the prevalence of MAP-positive patients presenting to our ED, which parallels statewide, domestic, and worldwide trends. Resource utilization has also changed, such as a lower rate of arrival by ambulance and hospital admissions. The lower proportion of blunt trauma and higher proportion of chest pain may reflect the shifting demographics of MAP users, as highlighted by an older patient population than in the past. Emergency clinicians will continue to be on the forefront of care for MAP patients presenting acutely to the ED for a myriad of medical, traumatic, and psychiatric issues. Toxicology screening of patients with suspected drug use and unexplained symptoms may be clinically and financially useful in this setting by decreasing laboratory and imaging testing, admission to the hospital, and length of stay. Unless there are major changes in focused law enforcement on MAP, penalties for production and possession, international border control, availability, pricing, and rehabilitation options for treatment of MAP addiction, the problem is predicted to continue its seemingly inexorable rise.

## Figures and Tables

**Table 1:** Demographics, mode of arrival, disposition, and coingestions of MAP-positive patients, 2016 versus 1996.

	2016	1996	% change	95% CI	*P*
	*n* (%)	*n* (%)			
Prevalence	638/20,203 (3.2)	461/32,156 (1.4)	1.8	1.5–2.1	<0.0001
Positive MAP screen	638/3013 (21.2)	461/3102 (14.9)	6.3	4.4–8.2	<0.0001
Age ± SD	41.6 ± 12.6	34.9 ± 8.5			<0.0001^†^
*Gender*					0.97
Male	409 (64.1)	295 (64.0)	0.1	−5.7–5.9	
Female	229 (35.9)	166 (36.0)	−0.1	−5.7–5.9	
*Race*					0.4
Caucasian	451 (70.7)	341 (74.0)	−3.3	−2.2–8.7	0.23
Hispanic	107 (16.8)	63 (13.6)	3.2	−1.2–7.5	0.15
African American	46 (7.2)	37 (8.0)	−0.8	−2.5–4.2	0.62
Asian/Pacific Islander	33 (5.2)	18 (3.9)	1.3	−1.4–3.9	0.31
Native American	1 (0.1)	2 (0.5)	−0.4	−0.4–1.6	0.21
*Insurance*					<0.00001
None/Self-pay	172 (27.0)	385 (83.5)	−56.5	51.3–61.2	<0.0001
MediCal/MediCare	396 (62.0)	56 (12.2)	49.6	44.7–54.5	<0.0001
HMO/MCO	70 (11.0)	20 (4.3)	6.7	3.4–9.8	0.0001
*Mode of arrival*					<0.00001
Ambulance	332 (52.0)	319 (69.2)	−17.2	11.3–22.9	<0.0001
Ambulatory	156 (24.4)	66 (14.3)	10.1	5.3–14.8	<0.0001
Police	121 (19.0)	56 (12.1)	6.9	2.4–11.2	0.002
Transfer	29 (4.6)	20 (4.4)	0.2	2.5–2.8	0.87
*Coingestion*					
Ethanol	79 (12.4)	92 (20)	−7.6	3.1–12.2	0.0006
Ethanol level (mg/dL)	148 ± 116.2	125 ± 32			0.07^†^
Cocaine	28 (4.4)	34 (7)	−2.6	−0.3–5.7	0.06
*Disposition*					<0.00001
Admit	263 (41.2)	268 (58.1)	−16.9	10.8–22.9	<0.0001
Discharge^*∗*^	144 (22.5)	89 (19.3)	3.2	−1.8–8.1	0.2
Psychiatric hold/transfer	223 (35.0)	63 (13.7)	21.3	16.2–26.2	<0.0001
Jail	8 (1.3)	41 (8.9)	−7.6	4.9–10.7	<0.0001

*∗* includes patients who eloped; ^†^Student's *t*-test. MAP: methamphetamine; HMO/MCO: health maintenance organization/managed care organization; CI: confidence interval.

**Table 2:** Presenting complaints of MAP-positive patients, 2016 versus 1996.

	2016	1996	% change	95% CI	*P*
	*n* (%)	*n* (%)			
Blunt trauma	78 (12.2)	152 (33.0)	−20.8	15.7–25.9	<0.0001
Altered LOC	185 (29.0)	108 (23.4)	5.6	0.2–10.9	0.04
Abdomen pain	54 (8.5)	58 (12.6)	−4.1	0.3–8.0	0.03
Suicide attempt/ideation	67 (10.5)	38 (8.2)	2.3	1.4–5.8	0.2
Chest pain	102 (16.0)	36 (7.8)	8.2	4.2–12.0	0.0001
Skin infection	45 (7.0)	28 (6.1)	0.9	−2.3–3.9	0.55
Penetrating trauma	30 (4.7)	20 (4.4)	0.3	−2.4–2.9	0.81
Miscarriage	7 (1.1)	8 (1.7)	−0.6	−0.9–2.4	0.4
Ingestion	47 (7.4)	8 (1.7)	5.7	3.2–8.2	<0.0001
Headache	23 (3.6)	5 (1.1)	2.5	0.6–4.4	0.009
Total	638	461			<0.0001

MAP: methamphetamine; LOC: level of consciousness; CI: confidence interval.
